# Unraveling the Role of Angiogenesis in Cancer Ecosystems

**DOI:** 10.3389/fonc.2018.00248

**Published:** 2018-07-02

**Authors:** Iratxe Zuazo-Gaztelu, Oriol Casanovas

**Affiliations:** Tumor Angiogenesis Group, ProCURE, Catalan Institute of Oncology – IDIBELL, Barcelona, Spain

**Keywords:** angiogenesis, angiogenic tumor ecosystem, sprouting angiogenesis, vasculogenesis, vasculogenic mimicry, intussusception, antiangiogenics

## Abstract

Activation of the tumor and stromal cell-driven angiogenic program is one of the first requirements in the tumor ecosystem for growth and dissemination. The understanding of the dynamic angiogenic tumor ecosystem has rapidly evolved over the last decades. Beginning with the canonical sprouting angiogenesis, followed by vasculogenesis and intussusception, and finishing with vasculogenic mimicry, the need for different neovascularization mechanisms is further explored. In addition, an overview of the orchestration of angiogenesis within the tumor ecosystem cellular and molecular components is provided. Clinical evidence has demonstrated the effectiveness of traditional vessel-directed antiangiogenics, stressing on the important role of angiogenesis in tumor establishment, dissemination, and growth. Particular focus is placed on the interaction between tumor cells and their surrounding ecosystem, which is now regarded as a promising target for the development of new antiangiogenics.

## Foundations of the Tumor Stromal Ecosystem

The simplistic view of a tumor as a conundrum of just mutant cells engaged in clonal expansion is currently evolving into a more holistic approach where tumors are regarded as organ-like structures (1, 2). Genetic deletion, overexpression, mutation, and translocation events certainly lead to the transformation of a normal cell into a malignant cell which will then undergo sustained proliferation. However, for neoplastic cell expansion and growth, the ability to handle the surrounding stroma to create a favorable ecosystem becomes imperative (3). Hence, the information enclosed in the rich and ever-changing tumor microenvironment is crucial for the understanding of antitumor drug sensitivity.

**Figure 1:**
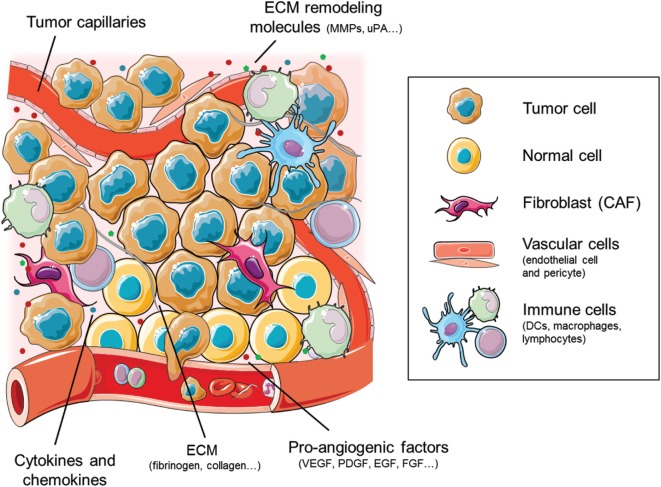
Cellular and molecular components of the tumor ecosystem that shape the tumor angiogenic landscape. The cellular components primarily consist of tumor and normal cells, together with the vascular endothelial and pericyte cells and the stromal fibroblasts [cancer-associated fibroblasts (CAFs)]. The immune cell compartment comprises mainly tumor-infiltrating macrophages, dendritic cells (DCs), and lymphocytes. Figure was created using Servier Medical Art according to a Creative Commons Attribution 3.0 Unported License guidelines 3.0 (https://creativecommons.org/licenses/by/3.0/). Simplification and color changes were made to the original cartoons.

The tumor microenvironment is formed by a tangled combination of both tumor and stromal cells, extracellular matrix (ECM), and secreted factors, thus perfectly fitting in the definition of an ecosystem (4, 5). Alteration of the gene expression of tumor cells provokes a disruption in the normal tissue homeostasis, favoring the secretion of certain molecules (cytokines, growth factors, etc.) that recruit stromal cells. Cells composing the tumor stroma are cancer-associated fibroblasts (CAFs), endothelial cells, pericytes, adipocytes, and immune cells, including monocytes, macrophages, lymphocytes, and dendritic cells (DCs), among others (Figure 1). These cells are enclosed in heterogeneously deposited ECMs and are affected by changing biophysical parameters including oxygenation and pH (6–9).

The insight into the dynamic action of the tumor ecosystem has improved exponentially over the last years, regarding the stroma as an integral part of tumor initiation, progression, and malignization. Stromal elements hold the key for prognostic and response predictive information. As such, therapeutic targeting of stroma-related processes are continually described. Tumor cells dwell in symbiosis with the rest of the body, mimicking and coopting several normal physiological processes on behalf of their surrounding stroma. Together with sustained proliferation and recruitment of immune cells, angiogenesis is one of the acknowledged promoters of tumor growth and survival (6, 10). In fact, tumor-associated vessels also contribute to dissemination of tumor cells by abetting their entry into the circulatory system and aiding in the generation of the pre-metastatic niche. In this review, we will further explore the role of angiogenesis as a key modulator inside the tumor ecosystem. To do so, we will first describe the different mechanisms responsible for tumor angiogenesis and we will focus later on the action of antiangiogenic drugs upon the stroma.

## Insight into the Angiogenic Tumor Ecosystem

To grow beyond a limited size, all solid tissues require a proper vasculature that grants oxygen, nutrients, and waste disposal. Since neoplasms are no exception to this rule, early activation of angiogenic processes is mandatory to sustain the deregulated proliferation of tumor cells. Apart from serving as nutrient, oxygen, and waste transport providers, vessels also facilitate dissemination of tumor cells to distant sites, promoting metastasis. Tumor angiogenesis is thus defined as the process of blood vessel creation, penetration, and growth in the tumor ecosystem.

The angiogenic program is switched on in response to hypoxia, which, together with the lack of nutrients, bolsters the expression of inflammatory signals and cytokines that recruit vascular cells for the tumor vessel plexus formation (11, 12). Early during tumor progression, hypoxia triggers the transcription of several genes that are key mediators of the angiogenic process, such as VEGF and PDGF (13). Mechanistically, activation of the angiogenic process involves the breakdown of the vascular ECM at different levels for subsequent endothelial cell invasion and tube formation (14). Apart from the role of tumor cells as principal secretors of endothelial cell promoters, the interplay with other stromal cells such as pericytes is also needed for neovessel stability.

For studying tumor angiogenesis, different approaches exist. A compilation of the currently used *in vivo, ex vivo*, and *in vitro* bioassays has been recently published as a collaborative work of some of the main experts in the angiogenesis field (15). Briefly, *in vivo* experimental models allow the study of mechanisms, kinetics, and dynamics in the context of a complex organism. The chorioallantoic membrane of a chicken embryo is used without graft rejection, making it easy and low cost to complete a drug testing assay (16, 17). However, vessel formation is difficult to assess in this model. Besides, zebrafish embryo model also has the translationality for tumor angiogenesis study. Due to its transparency, it allows easy imaging of the tumor angiogenic process (18). Among the existing animal models, mouse models are the ones that better mimic the complexity of human cancer as an evolutionary process while, at the same time, allow easy and cheap monitoring of the process. Even though subcutaneous xenograft induced angiogenesis is easy to visualize, orthotopic transplantation is better regarded as it considers the role of the tumor ecosystem. Currently used mouse models for are reviewed in Gengenbacher et al. (19).

Recently, outstanding advances in the *in vitro* and *in silico* development of tumor angiogenesis models have been made. *In vitro* approaches include the use of microfluidic cancer vasculature on-chip systems, whereas *in silico* models comprise mathematical processes that address tumor growth dynamics. Their progress and challenges are extensively reviewed by Soleimani and colleagues (20).

## Mechanisms Involved in Tumor Vessel Generation

Nearly 40 years after the studies that laid the foundations in the field (21), research in tumor angiogenesis has extensively matured, permitting the gathering of detailed knowledge over the processes that govern pathological vessel proliferation. Vessels are ordered tubular networks that permit transportation of nutrients, cells, and gases. Apart from providing nutrients, vessels function as carriers of instructive trophic signals needed for organ morphogenesis (22). Different types of vessels, including arteries, veins, and capillaries, are formed by a luminal side surrounded by a monolayer of endothelial cells. On the outside, following the basement membrane, vessels are covered by a layer of mural accessory cells composed of pericytes and vascular smooth muscle cells.

**Figure 2:**
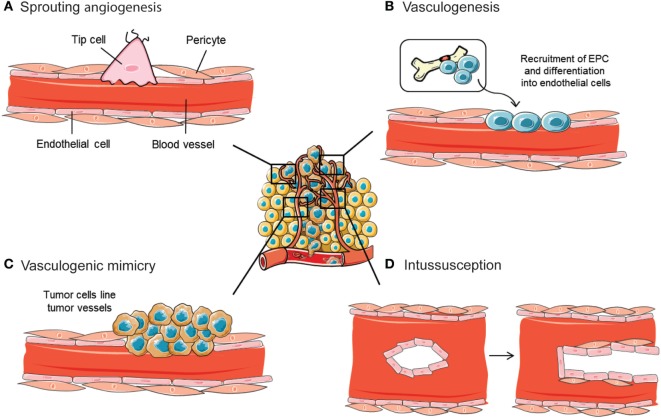
Mechanisms implicated in blood vessel formation. In the tumor ecosystem, blood vessels grow by sprouting angiogenesis **(A)**. In addition, less frequent neovascularization mechanisms include recruitment of bone marrow-derived endothelial progenitor cells (EPCs) **(B)**, intussusceptive microvascular growth **(C)**, and vasculogenic mimicry **(D)**. Figure was created using Servier Medical Art according to a Creative Commons Attribution 3.0 Unported License guidelines 3.0 (https://creativecommons.org/licenses/by/3.0/). Simplification and color changes were made to the original cartoons.

Archetypal mechanisms for neovascularization include vasculogenesis and sprouting angiogenesis (Figures 2A,B). Critical for the formation and remodeling of vessels during development, both mechanisms are reactivated during tumor progression. Vasculogenesis is defined as the *de novo* formation of blood vessels as a consequence of vascular progenitor cell differentiation, whereas sprouting angiogenesis stands for the formation of new vascular structures from a preexisting vessel network. Recently, the role of other less frequent vascular formation mechanisms during tumor growth has been described, including vasculogenic mimicry (VM) and intussusception (Figures 2C,D). Usually, neither of the mechanisms are mutually exclusive and even seem to act simultaneously in pathological neovascularization.

### Sprouting Angiogenesis

By far, sprouting angiogenesis is the best known angiogenesis-promoting mechanism used by tumor cells to induce their own vascularization from preexisting host capillaries (Figure 2A). A thorough interplay between ECM components, cells, and soluble factors, together with a sequence of well-defined steps, define sprouting angiogenesis (23). Destabilization of the endothelial–pericyte contacts, crucial for vessel integrity and maintenance of quiescence, initiates the process. Once the basement membrane that protects endothelial cells is destabilized, these cells undergo an endothelial–mesenchymal transition that triggers their proliferative, migratory, and invasive capabilities. Such activation further enhances the release of several proteases that induce ECM and basement membrane degradation, leading to guided migration and proliferation of vascular cells. The polarization of the moving endothelial cells eventually constitutes the vessel lumen, forming an immature blood vessel (24). An opposite mesenchymal–endothelial transition program is then activated to reverse the endothelial cells to their previous quiescent state. This latter step, known as vessel maturation, is characterized by the absence of angiogenesis, the recruitment of pericyte and mural cells, and the synthesis of a new basement membrane (25).

To engage the angiogenic process, endothelial cells need to follow a multistep specialization, which involves their plasticity in the angiogenic sprout and their following vascular guidance cue, that control the extension of the nascent vessel. The initiation of these morphogenetic events is marked by VEGF and Notch signaling pathways (26). Upon proangiogenic stimuli, sprouting endothelial cells change their phenotype toward an invasive and motile behavior, while activating protease secretion, cell–cell contact remodeling, and polarity reversal. The leading endothelial cells during the sprouting process are known as “tip cells.” Their response to VEGF signaling includes extending large filopodia that will allow guidance and sensing of the newly formed vessel, as well as the release of molecular signals that recruit stromal cells for vessel stabilization. On the other hand, endothelial cells can also evolve into highly proliferative cells located at the stalk of the angiogenic sprout. These “stalk cells” are responsible for tube and branch formation, thus assuring the expansion of the vascular structure in response to VEGF-A (27). Stalk cells also collaborate in the basement membrane deposition and establish junctions with adjacent cells to strengthen the integrity of the novel sprout (28).

By anastomosing with cells form adjoining sprouts, tips cells interconnect in vessel loops until their leading phenotype is switched off. The process ends with the reestablishment of quiescence, when proangiogenic signals decrease, a new basement membrane is formed, and VEGF levels dampen (29). During the transition between both states, endothelial cells gain a “phalanx”-like phenotype, becoming non-proliferative and immobile (30). Vessel stabilization and maturity are accomplished with lumen generation and pericyte recruitment along the new basement membrane, which leads to blood flow and perfusion initiation.

The functionality, correct extension, and morphology of the new vessels depend on the balance between stalk cell proliferation and tip cell guidance. Phenotypic specialization of endothelial cells in each of those types depends, in turn, on the balance between proangiogenic factors and endothelial proliferation suppressors (31). Inside the tumor ecosystem, this balance is shifted in favor of a proangiogenic milieu, thus generating a sustained sprouting angiogenic process that produces abnormal vascular structures.

### Vasculogenesis

The term “vasculogenesis” was conceived by Werner Risau, to define the physiological formation of the vascular plexus from the mesoderm as a consequence of angioblast differentiation (32). During tumor vasculogenesis, endothelial progenitor cells (EPCs) are mobilized and recruited in response to several chemokines, cytokines, and growth factors produced by tumor and stromal cells (Figure 2B). In particular, tumor cells produce a plethora of cytokines and proangiogenic factors, such as VEGF, that recruit bone marrow-derived DCs and induce their proliferation and differentiation (33). In hypoxic conditions, HIF is able to activate the transcription of VEGF, PDGF, stromal-derived factor 1 (SDF-1), and C-X-C chemokine receptor type 4 (CXCR4) (34). Studies with loss of function of HIF demonstrated an inhibition of EPC proliferation and differentiation. The contribution of vasculogenesis to tumor progression has also been demonstrated by knockout studies where some initiator molecules, such as inhibitors of differentiation factors, were genetically ablated. This approach provoked a disruption of tumor vascularization, angiogenesis blockade, and tumor growth impairment that was rescued by the restoration of the mobilization factors after bone marrow transplantation (35).

The first step of EPCs mobilization starts with the proangiogenic factor-mediated activation of the matrix metalloprotease 9 (MMP9) in the osteoblastic zone. Activated MMP9 proteolytically processes the membrane bound Kit ligand to its active soluble conformation. Kit is a stem cell-active migratory cytokine that induces migration and release of EPCs into the circulatory system (36). Once homed, EPCs are either incorporated into angiogenic sprouts or into the endothelial cell monolayer, aided by selectins and integrins (37). Endothelial cell maturation is substantially mediated by VEGF, which also contributes to vessel size establishment. Besides, EPCs share a paracrine mechanism that also triggers tumor angiogenesis by the release of proangiogenic molecules at the sites of neovascularization (38).

Depending on the experimental cancer model and the type of the tumor, vasculogenesis contributes to tumor vessel formation processes ranging from 0.1 to 50% of all vessels. As an example, the tumor ecosystem of hematopoietic and lymphoid tissues is more dependent on EPCs. Besides its role in primary tumor growth, vasculogenesis is also involved in dissemination and metastasis. SDF-1 produced by immune cells might attract EPCs to distant sites and once there spontaneously induce SDF-1 production, generating a gradient of this molecule that will serve as a chemoattractant of tumor cells. The interaction between SDF-1, secreted by EPCs, and its CXCR4 receptor, mainly expressed by tumor cells, would promote extravasation and development of the pre-metastatic niche (39). Moreover, the activation of MMP9 by EPCs is also related to an increase in tumor cell migration and invasion, confirming the role of vasculogenesis in metastatic niche formation (40).

### Vasculogenic Mimicry

Vasculogenic mimicry refers to the ability of some malignant cells to start the dedifferentiation process to adopt multiple cellular phenotypes, including endothelial-like properties (41) (Figure 2C). Those cells finally converge in *de novo* vasculogenic-like networks composed of red blood cells that are able to contribute to circulation (42). In this way, cells undergoing VM are able to reproduce the pattern of an early embryonic vascular plexus, providing the tumor ecosystem with an additional circulatory system independent of angiogenesis.

The process of VM was observed in highly invasive melanoma cells, whose phenotype reverted to an embryonic-like state and increased cell plasticity, including expression of endothelium-associated genes such as Ephrin-A2 and VE-cadherin (43). Release of ECM components, hypoxia, and activation of transmembrane metalloproteinases has been described as VM promoters (44). Although the occurrence of VM is relatively infrequent within tumors, it has been related to aggressive tumors, an increased risk of metastasis and poor prognosis (45).

### Intussusception

Vessel intussusception or intussusceptive microvascular growth (IMG) is defined as a developmental intravascular growth mechanism consisting of the splitting of preexisting vessels into two new vascular structures. This was first described in postnatal remodeling of lung capillaries (46) (Figure 2D). During intussusception, endothelial cell proliferation is not required, which ultimately makes it a rapid process that occurs within hours or minutes if compared with sprouting angiogenesis. Furthermore, IMG does not rely on endothelial cell proliferation, but it is rather a remodeling process of the endothelial cells that happens as a consequence of both their narrowing and volume increase. IMG is described to occur after sprouting angiogenesis or vasculogenesis, as a mean of expanding the capillary plexus without the need of a high-metabolic demand (47).

The “touching spot” between endothelial cells from opposite walls initiates the IMG process. To reinforce the transendothelial cell bridge, the endothelial bilayer is formed with cell–cell junctions and the interstitial pillar is formed. Pericytes and other mural cells are recruited to cover the interstitial wall, which is later widened, allowing endothelial cell retraction and the creation of two independent vessels (47). By using this mechanism, a large vessel is able to split into many smaller functional vessels. Although the precise mechanism underlying IMG is not fully described, alterations in blood flow dynamics, wall stress over pericytes, changes in shear stress on endothelial cells sensed by absence of CD31 and VEGF are some of the possible events that result in IMG initiation (48).

Intussusceptive microvascular growth has been reported in mammary, colorectal, and melanoma tumors (49). In human melanomas, a correlation between VEGF and intussusceptive angiogenesis was found, together with a higher number of intraluminal tissue folds (50). This scenario suggests that sprouting angiogenesis inhibition could stimulate IMG. Taking into account that intussusceptive angiogenesis only occurs in preexisting vascular structures, its most important contribution to tumor malignization is its ability to augment the number and complexity of tumor microvessel networks already created by other angiogenic mechanisms. Ultimately, the creation of new vessel structures also provides additional surface for further activation of sprouting angiogenesis.

## Role of Tumor Ecosystem in Promoting Angiogenesis

Inside the tumor ecosystem, tumor cells are the main producers of the proangiogenic molecules that switch on the angiogenic program. Among the molecules that regulate this process, PDGF, HGF, FGF, and, particularly, VEGF and its cognate receptors (VEGFRs) are the driving force, owing to their specific expression on tumor and endothelial cells. Nevertheless, other cells composing the tumor ecosystem also contribute to tumor angiogenesis and their role must be considered throughout an integrative approach (Figure 1).

### Cancer-Associated Fibroblasts

Cancer-associated fibroblasts normally originate from tumor or resident stroma, even though they can also differentiate from bone marrow precursors. While CAF-mediated secretion of proteases contributes to ECM degradation, CAFs also produce and deposit ECM, remarking a dual role for these cells in ECM remodeling. Besides, CAFs also secrete multiple angiogenic cues, participating in tumor growth and progression (51). Due to their primary localization at the leading edge of the tumor, where expanded vessel supply is demanded, the contribution to angiogenesis by stromal fibroblasts becomes crucial (52, 53).

One of the most important molecules secreted by stromal CAFs is VEGF-A, which was found to be induced in the stroma of both spontaneously arising and implanted tumors of genetically engineered mice with a reporter for VEGF-A (54). Actually, in ovarian carcinomas, most angiogenic growth factors are provided by CAFs rather than by malignant cells (55). CAFs also supply other factors such as angiopoietin-1 and -2, which are needed for neovascular stabilization (56).

### Immune Cells

The tumor ecosystem constitutes a crucible of heterogenous immune cell populations, resulting in tangled interactions between tumor cells and stroma. Immune cells have a remarkable role during the regulation of different aspects of tumor growth, such as modulation of angiogenesis and immune system evasion (57). Particularly, the contribution of macrophages, DCs, and mast cells is further explored in this section.

Tumor-associated macrophages (TAMs) represent one of the most abundant leukocyte population in the tumor ecosystem and their presence correlates with a reduction in survival in most tumor types (58). Regarding their phenotype, macrophages can be classified into the classically activated M1 and alternative activated M2 subsets. Whereas M2 macrophages show a proangiogenic phenotype, M1 macrophages have been described as antitumor effectors (59). TAMs often shift toward the M2 phenotype, becoming an important supplier of angiogenic cytokines and ECM remodeling molecules (60–62). Indeed, in different types of tumors, macrophage presence has been correlated with high vascularity (63, 64). Apart from the canonical signaling pathways, alternative proangiogenic molecules such as semaphorins and plexins have been also described as mediators of the macrophage–endothelial cell cross talk (65).

Dendritic cells, due to their potent antigen-presenting ability, are considered a critical factor in antitumor immunity (66). Nevertheless, defective myelopoiesis inside the tumor ecosystems renders DCs incompetent (67). A role for DCs in tumor angiogenesis has been described after the finding that immature DCs increased neovascularization in implanted tumor models, while depletion of DCs revoked angiogenesis (68).

Mast cells were found more than 30 years ago to be accumulated in tumors before the onset of angiogenesis, residing in close proximity to blood vessels (69). Those granulocytes participate in tumor rejection by IL1, IL4, IL6, and TNF-α production. However, mast cells also promote tumor growth by increasing the angiogenic supply, degradation of the ECM and immunosuppression (70). In detail, mast cells release angiogenic cytokines, such as VEGF, FGF-2, and TGF-β, among others (71).

### Vascular-Associated Components

Even though endothelial cells are the main players of the angiogenic tumor ecosystem, other components of the vascular system, such as platelets and pericytes, are also necessary for the proangiogenic switch. For instance, platelets, best known for their role in assisting the blood clotting process, have also been described as proangiogenic cells. Upon interaction with tumor cells, platelets are able to release VEGF from α granules (72, 73).

The contractile cells that surround the basement membrane of vessels are known as pericytes. In absence of angiogenesis, pericytes commonly express proteins such as PDGFRβ, NG2, and desmin and lack expression of α-SMA. Upon the activation of angiogenic signaling *via* PDGF, TGF-β, angiopoietin, and Notch, tumor pericytes loosen their attachment to the vessel, leading to a higher permeability of blood vessels (74, 75). Particularly, the recruitment of pericytes to the tumors highly depends on PDGF-B ligand production by endothelial cells (76, 77).

Nevertheless, the ultimate outcome of pericyte-derived signaling remains to be fully elucidated, since it seems to be context dependent. On the one hand, ectopic expression of PDGF-B in a mouse melanoma model increased tumor growth, indicating that a more stable and functional neovasculature was achieved through pericytes (78, 79). On the other hand, PDGF-B transfection into colorectal and pancreatic tumor cell lines inhibited tumor growth as a consequence of the angiostatic effect of recruited pericytes (80). Pericytes are also involved in the control of the metastatic spread of tumor cells (81). In fact, an increased rate of metastasis was described in a pancreatic neuroendocrine tumor mouse model genetically designed to be pericyte-poor. It remains to be elucidated whether their protective effect against metastasis is due to their active participation or as a consequence of their passive role as a physical barrier to extravasation.

### ECM and the Vascular ECM

The organization and composition of the matrix that supports the cells of the tumor ecosystem is essential for the regulation of angiogenesis. In fact, mice bearing alterations in ECM molecules such as collagen, laminin, and fibronectin exhibit vascular abnormalities (82). Vessel ECM is constituted by the basement membrane BM, which is mainly composed of collagen IV and laminin (83) and provides a broad binding surface for other ECM proteins, integrin receptors, and growth factors. Those interactions lead to the activation of many signaling pathways, such as PI3K, AKT, and MAPK, which are involved in adhesion, migration, invasion, and proliferation, thus contributing to tumor angiogenesis (84).

The interstitial matrix that surrounds the BM, which comprises collagen I, II, and III, as well as fibronectin and fibrinogen, also contributes to tumor angiogenesis. It primarily functions as a reservoir of regulatory molecules, such as angiogenic growth factors, cytokines, and proteolytic enzymes (85). Moreover, binding of VEGF to fibronectin has been found to enhance the activity of VEGF. Concomitantly, tumor and stromal cells produce proteolytic enzymes, such as MMPs, that release fragments with promigratory and proangiogenic properties (86), besides the activation of ECM-sequestered growth factors (87).

## The Angiogenic Switch in Tumorigenesis

In the absence of new vasculature, during the avascular phase, tumor growth is normally limited to no more than 1–2 mm^3^. Tumors obtain nutrients and oxygen from nearby blood vessels and angiogenic processes are not observed. The avascular tumors reach a stable state characterized by a balance between proliferation and apoptosis. To grow beyond the restricted size and sustain unlimited proliferation, tumors require their vascular network to be extended. This transition from this avascular state to the angiogenic phase is commonly known as “angiogenic switch” and occurs early during tumor progression (88). In pursuance of angiogenic activation, tumor cells need to undergo numerous genetic and epigenetic rearrangements that grant them the angiogenic potential for both tumor growth and latter metastasis. Indeed, a plethora of experiments have shown that the lack of a functional vascular network leads to tumor apoptosis or necrosis, reinforcing the importance of tumor vasculature for tumor thriving (89).

The angiogenic switch depends on a dynamic balance between positive (proangiogenic) and negative (antiangiogenic) factors controlling vascular homeostasis (90). Under physiological conditions, this balance is shifted toward negative regulation of angiogenic processes, thus maintaining the quiescence of the vasculature. Once tumor progression is started, different mechanisms, such as the loss of tumor suppressor genes and oncogene upregulation, revert this balance. During the first steps of tumorigenesis, high levels of strong angiogenic inducers, such as VEGF and FGF, are released to the tumor ecosystem. VEGF is regarded as the canonical angiogenesis initiator and has been found to be expressed in most types of cancer in response to different stimuli. Besides hypoxia, hypoglycemia, and growth factors, overexpression of the oncogene Myc produces a 10-fold increase in VEGF levels (91). Apart from VEGF, other proangiogenic molecules upregulated for the engagement of tumor angiogenesis are PDGF, EGF, TGF-β, FGF, MMPs, and angiopoietins.

Aiming at evading the ECM-associated endogenous inhibitors, tumor cells are able to further upregulate proangiogenic factors and even lose the expression of tumor suppressor genes such as p53 (92, 93). Moreover, tumor cell metabolism shifts and becomes highly acidic, as a consequence of the Warburg effect (94). The net increase in glucose consumption produces an abnormal lactic acid release that turns lowers extracellular pH (95). High levels of lactate have been correlated with EMT, dissemination, and metastases of several types of human cancer, such as melanoma and Lewis lung carcinoma (96–98). In detail, acidification further promotes angiogenesis through the increased expression of VEGF (99).

### The Hypoxic Tumor Ecosystem

Lack of oxygen inside the tumor occurs as an inevitable consequence of the rapid expansion of the tumor mass. Neoplasms have been generally described as highly hypoxic structures, bearing distorted, and abnormal vascular networks, inefficient in oxygen transportation (100). Hypoxia is known to upregulate proangiogenic inducers and endothelial–pericyte destabilizing molecules (Ang-2) and downregulate inhibitors. Furthermore, mobilization of bone marrow-derived precursor cells and recruitment of immune cells to the tumor ecosystem is also positively controlled by hypoxia (101). By changing the cytokine milieu, hypoxia can also induce an immunosuppressive microenvironment, allowing immune system evasion by cancer cells (102).

Hypoxia also produces a metabolic switch to apoptosis inhibition, anaerobic metabolism, increased invasiveness, EMT, and metastasis (103). A stem-like phenotype is induced concomitantly with the release of cytokines like IL-6. Consistently, hypoxia-driven expression of VEGF, MMPs, and ANGPTL4 is crucial for intravasation (104). In detail, ANGPTL4 expression disrupts vascular endothelial tight junctions and augments permeability, thereby altering transendothelial barriers (105).

## Contribution of Angiogenesis to Metastasis and Invasion

Aside from the role in primary tumor ecosystem maintenance, tumor angiogenesis enables tumor cell invasion and dissemination and favors the creation of new secondary tumor ecosystems at metastasized sites. VEGF-mediated stimulation of blood and lymphatic endothelial cells provides a wide vascular area for intravasation of tumor cells, apart from increasing vascular permeability. In tumor endothelial cells, VEGF upregulates protease secretion, contributing to basement membrane degradation, and increasing the expression of molecules that mediate in tumor–endothelial cell interactions (106).

Other stromal cells also participate in the angiogenic-driven metastasis process. Pericytes covering tumor vessels are more loosely attached to endothelial cells, affecting endothelial cell survival, and increasing the number of intercellular gaps that permit easy access for tumor cell intravasation (81, 107). As a consequence of the increased vascular leakiness, passive escape of tumor cells is highly induced (108).

## Blocking Vessels in the Ecosystem

Fighting neovascularization to halt tumor progression has become a critical step of the long-established theory of angiogenic activation for tumor growth. In fact, more than 40 years have passed since tumor angiogenesis inhibition was first introduced as a potential therapeutic strategy (21, 109). Since then, many drugs targeting tumor vascularization have proven successful in the treatment of different tumors. Such is the case for the first FDA-approved angiogenesis inhibitors sunitinib (Sutent^®^) and bevacizumab (Avastin^®^), which demonstrated promising results in the treatment of kidney and colorectal cancers (110, 111).

Currently, using standard chemotherapy alone for cancer treatment has proven inefficient due to low selectivity of tumor cells, producing toxicity in normal tissues with high-proliferation rates (e.g., bone marrow, hair follicles, and gastrointestinal tract). Besides, tumor cells become resistant, whereas the abnormality of tumor vasculature impairs efficient drug delivery (112). On the contrary, with thousands of people being treated with VEGF inhibitors around the world, antiangiogenic targeting surely serves as an example of specific tumor ecosystem disruption for efficient cancer treatment.

There are different reasons underlying the success of tumor vascular targeting, involving both tumor and stromal cell interplay. First, the concept that tumors are dependent on multiple factors extrinsic to themselves, so rendering them without a functional vasculature that delivers oxygen and nutrients should kill them. Second, stromal cells, unlike neoplastic cells, are genetically more stable, being less likely to develop resistance to therapy. This makes angiogenesis a really attractive target for drug development. Third, tumors have always been described as highly vascular structures, meaning that anti-vascular targeting could be aimed at the treatment of a wide range of solid tumors (113, 114).

**Figure 3:**
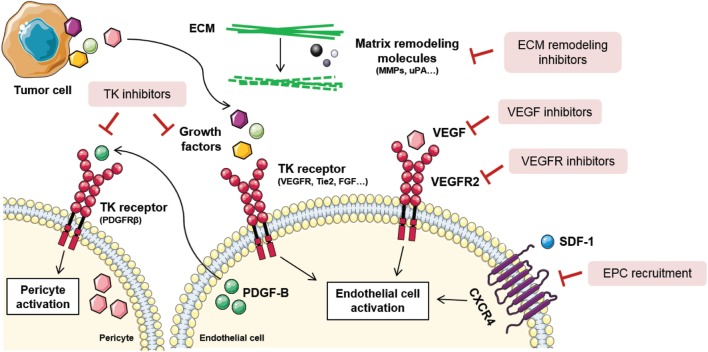
Tumor angiogenesis inhibition strategies. Due to the complexity of tumor angiogenesis, it can be inhibited at different levels. Direct vessel signaling inhibition approaches include VEGF ligand inhibitors, VEGFR receptor inhibitors, and other growth factors inhibitors released by stromal or tumor cells. Other examples are tyrosine kinase (TK) inhibitors, that block endothelial and pericyte cell activation, thus blocking their proliferation, migration, and survival. Novel antiangiogenic strategies are directed toward endothelial progenitor cell (EPC) recruitment inhibition, *via* stromal-derived factor 1 (SDF-1)/C-X-C chemokine receptor type 4 (CXCR4) signaling blockade, and extracellular matrix (ECM) remodeling inhibition. Figure was created using Servier Medical Art according to a Creative Commons Attribution 3.0 Unported License guidelines 3.0 (https://creativecommons.org/licenses/by/3.0/). Simplification and color changes were made to the original cartoons.

Taking into account the abundance of mechanisms involved in tumor angiogenesis, blood vessel formation processes can be inhibited at many different levels (Figure 3). Actually, distinct types of compounds, such as antibodies and small molecules, have been developed as antiangiogenic drugs. Production of antibodies presents some disadvantages for the pharma companies regarding the expensive requirement of mammalian cell production systems, dependence on disulfide bonds for stability, overcoming the tendency to aggregation, and low expression yields. Consequently, other promising molecules such as small globular proteins, aptamers, and peptides are currently being investigated (115). Noteworthy, not all antiangiogenic compounds have the same cellular effects nor the same therapeutic relevance. The main effects of angiogenic inhibitors can be classified according to their effects on: inhibition, regression, or normalization of tumor blood vessels. In this section, some of the main mechanisms to inhibit vascular malignization will be highlighted.

### Direct Vessel Signaling Inhibition

Endothelial cell activation is commonly initiated upon stimulation of tyrosine kinase (TK) receptors by growth factors. As previously stated, VEGF is the most important growth factor involved in tumor angiogenesis, and its inhibition influences endothelial cell survival, growth, migration, blood flow, and stromal cell recruitment (116, 117). Some of the VEGF-inhibiting approaches imply neutralization of the ligand or the receptor by specific antibodies, soluble receptors, small-molecule inhibitors of TK phosphorylation, and the direct inhibition of its intracellular signaling pathway (Figure 3). Thus far, 10 molecules that target VEGF or VEGFR have been approved for the treatment of various malignancies (118).

Since TK receptors are expressed both in tumor and vascular cells, TK inhibitors (TKIs) are regarded as a useful drugging strategy for their potentially dual effect (Figure 3). They are capable of blocking tumor cell proliferation and proangiogenic signaling simultaneously (119). However, the efficacy of TKIs varies depending on the different expression levels of the targeted ligands and effectors depending on the tumor type. Some strategies include compounds that block the binding site of the ATP in the TK receptor, causing the blockade of the receptor. Other TKIs aim at preventing the binding of the TK ligand with antibodies that block the growth factor or the binding site of the receptor (120).

The best known TKIs that block VEGFR and PDGF signaling are sorafenib, sunitinib, and pazopanib. Sorafenib is a synthetic compound that inhibits both Raf signaling, involved in cell division and proliferation, and VEGFR-2 and PDGFRβ signaling, modulators of angiogenesis (121). Its use is approved in the treatment of hepatocellular, thyroid, and renal cell carcinomas. Similarly, sunitinib is a TKI that, apart from blocking VEGFR-2 and PDGFRβ, is able to inhibit c-kit. The FDA approved the use of sunitinib for the treatment of imatinib-resistant gastrointestinal stromal tumor and renal cell carcinoma (122). Recently, anti-VEGFR2 antibody ramucirumab has received the FDA approval for second-line gastric cancer treatment (123). Another example includes pazopanib, a VEGFR-1, -2, -3, c-kit, and PDGFR inhibitor, approved for renal cell carcinoma and soft tissue sarcoma (124).

### Novel Antiangiogenic Approaches

#### Vascular Ecosystem Inhibition

Considering the contribution of EPCs to tumor angiogenesis and metastasis, blocking of EPC recruitment is a recently explored strategy for new blood vessel and metastatic niche abrogation (125) (Figure 3). To achieve so, specific targeting of molecules involved in EPC homing and recruitment from the bone marrow is an interesting approach. SDF-1/CXCR4 signaling axis is the main regulator of EPC mobilization and, as such, antagonists and antibodies against CXCR4 have been proposed (126). The action of these compounds is based on their ability to prevent the chemokine gradient that permits the homing of EPCs to the tumor ecosystem. Besides, VEGF is also a key modulator of EPC recruitment and preclinical studies have shown that VEGF blockade negatively modulates EPC-driven vasculogenesis (127).

Given that interactions between cells composing the tumor ecosystem and their surrounding ECM are crucial for angiogenesis regulation, modifying the structural and biochemical properties of the stroma should also impair vessel growth (128) (Figure 3). Among all the molecules that compose the ECM, MMPs are critically relevant for angiogenesis and tumor invasion, as demonstrated by genetic ablation studies where their absence impeded angiogenic tumor growth (129). In this context, tissue inhibitors of MMPs, together with synthetic inhibitors of serine proteases, such as urokinase type plasminogen activator, are regarded as potential antiangiogenics (130). Importantly, there are many endogenous angiogenesis inhibitors composing the ECM that are inactivated during the angiogenic switch. Many laboratories are trying to reproduce these natural angiogenesis inhibitors that act through binding αvβ3 and β1 integrins in endothelial cells. Some examples include arrestin, canstatin, and tumstatin (131).

Since the combination of immune checkpoint inhibitors with VEGF targeted agents shows a strong preclinical rationale, several undergoing studies are exploring its potential clinical exploitance [as reviewed in Ref. (132)]. As an example, a study combining bevacizumab with anti-CTLA4 in melanoma patients showed an increased infiltration of immune cells and extensive morphological changes of CD31 + endothelial cells (133). In a recent study, the use of axitinib, a multireceptor inhibitor that targets VEGFR, PDGF, and c-kit, demonstrated a depletion of mast cells together with an improved T-cell response, pivotal for the therapeutic efficacy (134).

#### Vessel Normalization

In comparison with physiologic tissue vasculature, tumor vasculature is characterized by aberrant, dilated, disorganized, and tortuous blood vessels. Lack of pericyte association and vascular immaturity produce excessive permeability, increased hypoxia, and poor perfusion, resulting in decreased antitumor treatment efficacy. For instance, chemotherapeutic drugs and immunotherapies are not able to reach all regions of the tumor (135, 136). To overcome this challenge, combination of antitumor treatments and low doses of vascular targeting agents are used. Careful dosage of antiangiogenics are able to restore normal levels of angiogenic signals in different types of tumors, provoking decreased permeability by recruiting pericytes and tightening cell–cell junctions (137). This phenomenon is known as “vascular normalization.”

Benefits of vascular normalization have been observed in different types of tumors. The combination of bevacizumab, together with chemotherapy, produced a positive outcome in a subset of breast cancer patients (138). Furthermore, combined inhibition of VEGFR and angiopoietin-2 improves survival of mouse glioblastoma tumor models, by increasing vessel normalization and reprogramming TAMs (139). Another example of the benefits of vessel normalization include the use of trebananib, a fusion protein that inhibits angiogenesis by blocking binding of angiopoietin-1 and -2 to Tie 2 receptor. In a recent study, combination of trebananib and chemotherapy demonstrated benefits in progression-free survival in epithelial ovarian cancer patients (140).

## Conclusion

Far ahead from the traditional idea that neoplasms are merely characterized by the tumor cells, tumors are now regarded as a heterogeneous association of both tumor and stromal cells that contribute in an interconnected fashion to malignant progression. The tumor ecosystem remains a bustling interchange of tumor cells, secreted molecules, and native tissue elements that, acting together, control the balance toward a proangiogenic program activation. In this way, the correct interaction between the components of the tumor ecosystem is critical for the success of the malignant lesion. Tumor stroma acts as a co-director for the development of vascularized growing mass, becoming the rationale driving the development of new antitumor therapies with antiangiogenic drugs.

Several years after the establishment of tumor angiogenesis as a cancer hallmark, the clinical exploitation of antiangiogenic therapies has reached a certain level of maturity (6). From the archetypal sprouting angiogenesis to describing less known mechanisms such as VM, the understanding of angiogenic mechanisms has become imperative for successful therapeutic targeting. The focus on the importance of these processes and the achievements in the clinical setting are reflected in the increasing number of drugs available to target angiogenesis mediators.

Undoubtedly, the normalization of the tumor ecosystem is an important new aspect for cancer treatment. Even though the tumor microenvironment holds many different cell types and components, the severity of the disease can be reduced by using a single effective drug, as demonstrated with antiangiogenics. Based on this observation, the combination of different therapies targeting different stromal components, together with traditional antitumor agents, could hold the key to impair cancer progression. Despite the rapid progress achieved in tumor ecosystem targeting, only a modest clinical success has been so far observed (141). Ongoing studies in the field which focus on studying the tumor ecosystem from an integrative point of view bear the potential to significantly control tumor angiogenesis and broaden the spectrum of current anticancer treatments.

## Author Contributions

Both IZ-G and OC have written, revised, and compiled this review.

## Conflict of Interest Statement

OC declares that has been economically compensated with his assistance to advisory boards and conferences from Novartis, Pfizer, Ipsen, and Teva. Apart from this, there is no conflict of interest that could be perceived as prejudicing the impartiality of the research reported.
